# Marine Fish Primary Hepatocyte Isolation and Culture: New Insights to Enzymatic Dissociation Pancreatin Digestion

**DOI:** 10.3390/ijerph18041380

**Published:** 2021-02-03

**Authors:** Neusa Figueiredo, Beatriz Matos, Mário Diniz, Vasco Branco, Marta Martins

**Affiliations:** 1 MARE—Marine and Environmental Sciences Centre, Departamento de Ciências e Engenharia do Ambiente, NOVA School of Science and Technology (FCT NOVA), 2829-516 Almada, Portugal; nl.figueiredo@campus.fct.unl.pt (N.F.); bi.matos@campus.fct.unl.pt (B.M.); 2 UCIBIO—Applied Molecular Biosciences Unit, NOVA School of Science and Technology (FCT NOVA), 2829-516 Almada, Portugal; mesd@fct.unl.pt; 3 Research Institute for Medicines (iMed.ULisboa), Faculty of Pharmacy, Universidade de Lisboa, 1649-003 Lisboa, Portugal; vasco.branco@ff.ulisboa.pt

**Keywords:** primary hepatocytes, in vitro assays, *Sparus aurata*

## Abstract

Primary cell cultures from wild organisms have been gaining relevance in ecotoxicology as they are considered more sensitive than immortalized cell lines and retain the biochemical pathways found *in vivo*. In this study, the efficacy of two methods for primary hepatocyte cell isolation was compared using liver from two marine fish (*Sparus aurata* and *Psetta maxima*): (i) two-step collagenase perfusion and (ii) pancreatin digestion with modifications. Cell cultures were incubated in L-15 medium at 17 ± 1 °C and monitored for up to six days for cell viability and function using the trypan blue exclusion test, MTT test, lactate dehydrogenase (LDH) activity, and ethoxyresorufin O-deethylase (EROD) activity after Benzo[a]Pyrene exposure. The results showed significant differences between the number of viable cells (*p* < 0.05), the highest number being obtained for the pancreatin digestion method (average = 4.5 ± 1.9 × 10^7^ cells). Moreover, the hepatocytes showed solid adherence to the culture plate and the rounded shape, changing into a triangular/polygonal shape. The cell viability and function obtained by pancreatin digestion were maintained for five days, and the EROD induction after exposure to the B[a]P showed that cells were metabolically active. This study shows that the optimized pancreatin digestion method is a valid, cost-effective, and simple alternative to the standard perfusion method for the isolation of primary hepatocytes from fish and is suitable for ecotoxicological studies involving marine pollutants, such as PAHs.

## 1. Introduction

Aquatic ecosystems, from coastline to open ocean, are widely impacted by a countless number of xenobiotics (e.g., heavy metals, persistent organic pollutants (POP), pharmaceuticals (e.g., triclosan), endocrine-disrupting chemicals (EDCs), and plastic wastes and their related chemicals (e.g., BPA, phthalates)) [1,2,3,4,5,6,7,8,9,10]. The presence of these xenobiotics in both water and sediments eventually results in ecotoxicological effects in aquatic organisms, from algae to fish [3,5,6]. For instance, exposure to polycyclic aromatic hydrocarbons (PAHs), such as Benzo[a]Pyrene (B[a]P), is known to produce carcinogenic effects following bioactivation by cytochrome P450 (CYP) and epoxide hydrolases [4].

Traditionally, in aquatic toxicology, the toxic effect of xenobiotics is mostly evaluated using in vivo models (e.g., algae, such as *Pseudokirchneriella subcapitata*; invertebrates, such as chironomids or daphnia; and fish, such as rainbow trout, fathead minnow, stickleback, medaka, or zebrafish) [11]. Besides being highly costly and time-consuming, this approach requires a substantial number of animals [12,13], which creates pressure for the replacement, reducing, and refinement (3Rs principle) of in vivo tests, particularly when the research goal is focused on understanding toxicity mechanisms. In this context, in vitro cell cultures offer advantages to the in vivo approach, as cells in culture retain the basic characteristics of the in vivo conditions [14,15] while allowing the precise control of physicochemical conditions (pH, temperature, osmotic pressure, and O_2_ and CO_2_ tension) and physiological conditions (such as hormone and nutrient concentrations) of the experiment, reducing the variability of results [15]. Furthermore, the costs and time expended are greatly diminished, as the maintenance of cell cultures is far less demanding than conducting assays with live fish.

The main limitation to the in vitro approach, especially when using immortalized cell lines, is the genetic and phenotypic instability that may introduce variability from one passage to another and the lack of environmental relevance, which compromises the effective extrapolation to whole organisms [16]. However, this can be partially overcome by the usage of freshly isolated primary cultures from species with environmental relevance. These cultures are a mixture of several cell types obtained from a piece of minced or enzyme-dispersed tissue, thus retaining the characteristics of their source tissue. In particular, in research using hepatocytes, the maintenance of many in vivo characteristics is the most important factor. For instance, Segner and Cravedi [17] found that primary hepatocytes express stable levels of phase I and phase II enzymes, such as CYP1A1, an important factor for studies of metabolically activated contaminants [18,19,20,21].

The critical point of using primary hepatocyte cultures is to obtain a viable culture that will survive and be functional long enough for toxicological assessment. The low viability and cell membrane damage associated with simple mechanical or enzymatic digestion methods have discouraged their usage. At the expense of these methods, the collagenase perfusion method became the classic and routinely used method for the isolation of primary hepatocytes by producing a higher number of hepatocytes, which are able to maintain vitality and function for a prolonged period [22]. Two-step collagenase perfusion was originally described by Berry and Friend [22] for the rat liver, but Birnbaum et al. [23] adapted it for the fish liver as well [24]. The authors Baksi and Frazier [22] and Pesonen and Andersson [21] revised the properties and usage of fish primary cell cultures and, even now, a considerable number of studies are still reporting the application of these cultures as a good alternative for the replacement of aquatic organisms [14,19,25,26,27,28,29]. However, most of these studies used freshwater species models, and there is a lack of information regarding the isolation and culture conditions for marine fish hepatocytes. Moreover, perfusion requires skillful handling of procedures, namely in the cannulation step, which may undermine the success of the culture.

Therefore, this study aims to optimize an effective and simple procedure for the isolation and maintenance of viable primary hepatocytes from marine fish, which can be used for ecotoxicological assessment of marine pollutants. To achieve this goal, two marine species with ecological and economic relevance, *Sparus aurata* and *Psetta maxima*, were used to isolate primary hepatocytes by two different methods: two-step collagenase perfusion and pancreatin digestion. The viability and functionality of the isolated hepatocytes were assessed under different culture conditions, including 3-(4,5-dimethylthiazol-2-yl)-2,5-diphenyltetrazolium bromide (MTT), lactate dehydrogenase (LDH), and ethoxyresorufin O-deethylase (EROD) assays.

## 2. Materials and Methods

### 2.1. Fish Acclimatization

The turbot (*Psetta maxima*, Linnaeus, 1758) and gilt-headed seabream (*Sparus aurata*, Linnaeus, 1758) weighing 46.8–75.1 g and 186.7–271.3 g, respectively, were acquired from a local aquaculture farm (Aquinova-Mira and Setúbal, Portugal, respectively). *P. maxima* is a bottom-dwelling marine fish, and *S. aurata* is the most economically important pelagic marine sparid fish species cultured along the Mediterranean coast and has been used in ecotoxicological studies [5,6,30,31,32,33]. The fish were transported to the MARLab laboratories at the Department of Environmental Sciences and Engineering (FCT NOVA) and maintained in controlled conditions (17 ± 1 °C, 34‰ salinity, with a regular photoperiod set at 12 h light/12 h dark). During this time, the fish were fed with standard commercial chow (as used by the fish supplier) twice a day.

### 2.2. Primary Hepatocyte Cell Isolation

All animals were randomly collected from the tanks, evaluated regarding the general health status, measured for total weight, and irreversibly anesthetized with an aqueous solution of 2-phenoxyethanol (0.25 ppm) [34]. After disinfection with 70% alcohol, a longitudinal incision from the anus to the gills was performed to expose the liver. Internal organs were observed for their general condition, and fish with excessively fat liver or alterations in tissue consistency were excluded from the procedure.

**Figure 1:**
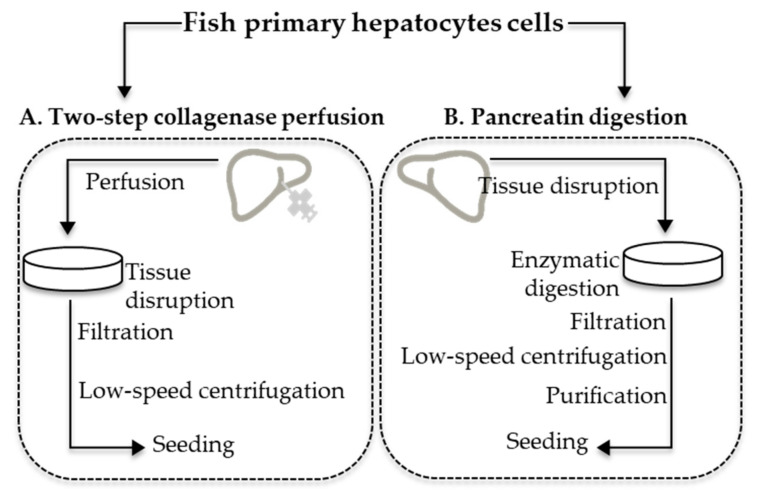
Primary fish hepatocytes isolation methods applied: (**A**) two-step collagenase perfusion and (**B**) pancreatin digestion.

**Figure 2:**
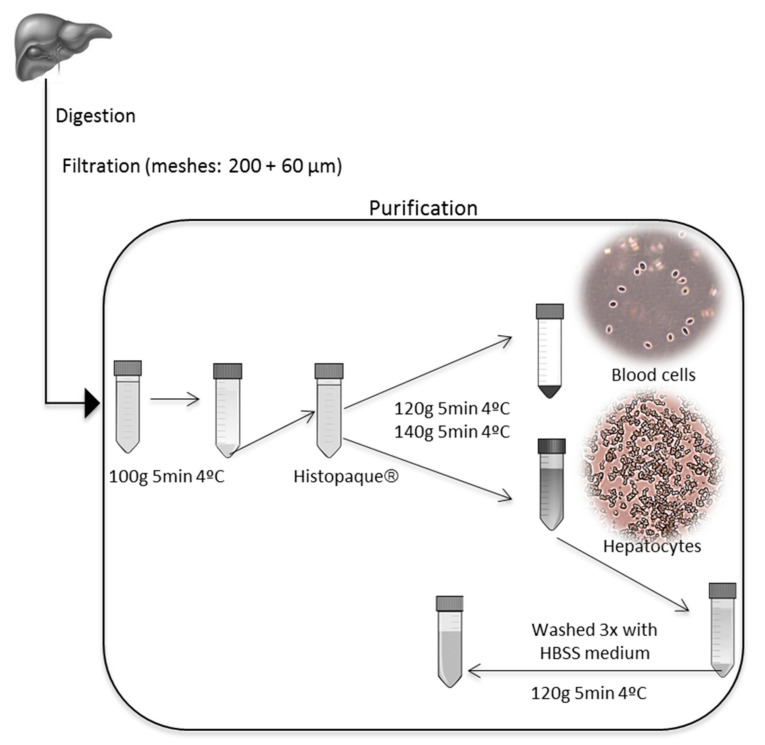
Pancreatin digestion method by Yanhong et al. [24] with modifications to increase culture purity.

The primary hepatocytes were isolated using the two-step collagenase perfusion and pancreatin digestion methods, according to Ferreira et al. [19] and Yanhong et al. [24], respectively, with modifications (Figure 1 and Figure 2) as subsequently described. In total, 15 fish were used for both procedures.

#### 2.2.1. Two-Step Collagenase Perfusion

A cannula (24G) was inserted in the portal vein, and the liver was first perfused for 20 min with solution A (176 mM NaCl, 4.82 mM KCl, 0.44 mM KH_2_PO_4_, 3.6 mM NaHCO_3_, 0.35 mM Na_2_HPO_4_.2H_2_O, 10 mM HEPES, 5 mM Na_2_-EDTA, pH 7.6) at a flow rate of 10 mL min^−1^. The second perfusion step was performed for 15 min with solution B (solution A without Na_2_EDTA, with 2.5 mM CaCl_2_ and 0.02 mg mL^−1^ collagenase IV) at the same flow rate (10 mL min^−1^). Following the two perfusions, the liver was removed and transferred to a chilled dish containing solution C (solution B without collagenase IV and with 1% BSA) for 30 min and mechanically disrupted by using sterile stainless-steel scissors. The liver fragments were then filtered twice through a stainless-steel 200 and 64 µm mesh, and the cells were collected by low-speed centrifugation (100× *g*, 5 min, 4 °C). The cell pellet was washed three times in cooled nonsupplemented Hank’s balanced salt solution (HBSS, Lonza) and then resuspended in HBSS medium supplemented with 5% fetal bovine serum (FBS), penicillin (10 U mL^−1^), streptomycin (10 μg mL^−1^), and amphotericin (0.025 μg mL^−1^). The cell yield was counted in a Neubauer chamber and their viability was examined by the trypan blue exclusion assay. The percentage of viable cells was calculated as (A − B) / A × 100, with A being the average of total cells and B the average of nonviable cells.

After opening the abdomen, the liver was: (A) perfused in situ, digested with collagenase, and disrupted with stainless-steel forceps; (B) cut into small pieces, clipped into fragments, and digested with pancreatin digestive juice. The digested fragments were filtered through stainless-steel meshes (200 µm and 60 µm), and the cells were collected by low-speed centrifugation (100× *g*).

#### 2.2.2. Pancreatin Digestion

The liver was removed from the abdomen, weighed, and placed in a cool dish containing sterile dissection balanced salt solution (DBSS) (126 mM NaCl, 4.82 mM KCl, 1.5 mM KH_2_PO_4_, 6.1 mM Na_2_HPO_4_, 21.9 mM HEPES, pH 7.4). The liver was then cut into 1.0–2.0 mm^3^ pieces, washed three times with cool sterile DBSS, and clipped into fragments. The preheated (37 °C) pancreatin digestive juice (126 mM NaCl, 4.82 mM KCl, 1.5 mM KH_2_PO_4_, 6.1 mM Na_2_HPO_4_, 0.54 mM Na_2_-EDTA, 0.1% pancreatin) was added to the dish to digest the fragments for 30 min. During the digestion, the fragments were blown gently with a sterile Pasteur pipette. The digested fragments were then filtered through a stainless-steel mesh (200 µm and 60 µm, respectively) and collected by low-speed centrifugation (100× *g*, 5 min, and at 4 °C). The pellet was purified with Histopaque^®^ (Sigma) [35] (120× *g*, 5 min 4 °C and 140× *g*, 5 min 4 °C). The hepatocytes were collected from the supernatant, washed three times with nonsupplemented HBSS, and then resuspended in HBSS medium supplemented with 5% FBS, penicillin (10 U mL^-1^), streptomycin (10 μg mL^−1,^) and amphotericin (0.025 μg mL^−1^). The cell yield was counted in a Neubauer chamber, and viability was examined by the trypan blue exclusion assay. The percentage of viable cells was calculated as described in Section 2.2.1.

### 2.3. Seeding Fish Primary Hepatocytes

To establish marine fish primary hepatocyte cultures, the isolated hepatocytes showing ≥85% viability [36] were cultured in a complete cell medium consisting of L-15 medium, supplemented with 5% FBS, penicillin (10 U mL^−1^), streptomycin (10 μg mL^−1^), and amphotericin (0.025 μg mL^−1^), in 96-well microplates (10^4^ cells/well), 24-well microplates (10^5^ cells/well), 6-well microplates (10^6^ cells/well), and 35 mm cell culture dish (10^6^ cells/well), with a distinct growth surface coating. The growth surface of each type of plate was previously coated with 0.01% poly-L-lysine solution (Sigma-Aldrich) and Biocoat Collagen I (Corning). In parallel, plates without treatment were used as controls. The cell cultures were allowed to attach for 24 h (hereafter defined as T_0_ cells) and maintained in an incubator at 17 ± 1 °C (the fish optimal growth temperatures). The medium was changed every 2 days.

### 2.4. Monitoring of Fish Primary Hepatocyte Cultures: Morphology, Viability, and Functionality

This assessment was performed after 24 (T_24_), 48 (T_48_), 72 (T_72_), 96 (T_96_), and 144 h (T_144_) (6 days) of cell attachment (T_0_), depending on the endpoint.

#### 2.4.1. Cell Morphology

Cultured hepatocytes in 35 mm dish cultures were monitored daily under inverted light microscopy (Olympus) (10× and 20× objectives) to observe the morphological changes over time (i.e., from single rounded cells to aggregate polyhedral morphology typical of hepatocytes) [37].

#### 2.4.2. Cell Attachment and Viability

Cell attachment and viability were assessed at T_24_. Briefly, after the removal of culture media from culture plates (0.01% poly-L lysine, collagen I, and without coating), the primary hepatocytes were washed with sterile 1× PBS and detached with trypsin (0.25% trypsin–EDTA, Sigma). Cell detachment was observed under the microscope and an aliquot (50 µL) of the cell suspension was taken, and cells were counted in a Neubauer chamber by the trypan blue exclusion assay. Cell viability was expressed relative to the T_0_ cell number.

#### 2.4.3. Cell Viability by MTT

The MTT assay was performed using cell cultures at T_0_, T_24_, T_48_, T_72_, T_96_, and T_144_ and according to the methodology described by Mosmann and modified by Carvalho et al. [38,39]. Briefly, the MTT solution (at a final concentration of 400 µg mL^−1^ per well) was added to 96-well plates prepared previously with 104 cells/well followed by incubation at 16 or 18 °C (depending on fish species) for 4 h. Following the incubation period, the medium with the MTT was removed and the formazan crystals dissolved using a 150 μL/well 4:1 mixture of dimethyl sulfoxide (DMSO)/glycine buffer (50 mM glycine, 50 mM sodium chloride/NaOH, pH 10.5) by shaking for 20 min at 200 rpm. The absorbance was measured at 550 nm on a microplate reader. Cell viability was expressed as the percentage of T_0_ cells.

#### 2.4.4. Detection of LDH in the Supernatant

Lactate dehydrogenase (LDH) release was used as an indicator of hepatocyte mortality. This assay was performed using the culture medium collected at T_0_, T_24_, T_48_, T_72_, T_96_, and T_114_. After the collection of the medium from 24-well plates previously cultured with 10^5^ cells/well, the medium was centrifuged at 4 °C for 10 min (12,000 g) to remove floating cells. The LDH activity was measured using the Pierce LDH cytotoxicity assay kit (Thermo Scientific, USA), according to the manufacturer’s instructions. The absorbance of each sample was read at 490 nm and 680 nm using a microplate reader. A positive LDH control (from the kit) and negative control (L-15 medium without cells) controls were run in parallel.

#### 2.4.5. Ethoxyresorufin O-Deethylase (EROD) Activity

EROD (CYP1A) activity was measured according to the method adopted for the 24-well plate described by Ferreira et al. [18]. Briefly, after 24 h and 48 h of exposure to 0.1 µM, 0.5 µM, and 1 µM B[a]P, the medium was removed, and the cells washed once with cold PBS. EROD activity was measured by adding a EROD reaction mixture containing 1 mg/mL BSA, 5 μM 7-ethoxyresorufin (7-ER), and 0.5 mM NADPH and incubated for 30 min in the dark at 37 ± 1 °C. Fluorescence was measured at 560 nm excitation and 610 nm emission. EROD activity was calculated based on the resorufin standard curve obtained for each independent experiment. After EROD measurement, total protein concentration was determined in the same well by the Bradford method with BSA as a standard.

### 2.5. Data Analysis

Statistical significance of results was evaluated with the software Statistica 8.0 Statsoft, 2007 (Tulsa, USA) by applying the nonparametric Mann–Whitney *U* test to compare two independent variables or the parametric *t*-test for independent variables, depending on the observation of parametric test assumptions, i.e., normality of distribution (Shapiro–Wilk’s test) and homogeneity of variances (Levene’s test). All data were expressed as means ± SE of more than three independent experiments, and statistical differences were established as significant at *p* < 0.05 and very significant *p* < 0.01.

## 3. Results

### 3.1. Cell Yield and Viability

The cell yield obtained from *S. aurata* liver ranged from 1.04 × 10^7^ to 2.47 × 10^7^ for the two-step collagenase perfusion method and 3.3 × 10^7^ to 7.31 × 10^7^ for the pancreatin digestion method (Table 1), and the cell viability ranged from 70.7 to 72.8% for the two-step collagenase perfusion method and 95.1% to 100% for the pancreatin digestion method (Table 1). The cell yield obtained for *P. maxima* liver by pancreatin digestion ranged from 1.08 × 10^7^ to 3.44 × 10^7^ (Table 1). The small size of livers (average = 0.47 ± 0.19 g) made it difficult to perform the two-step collagenase perfusion method in this species.

The recommended work viability is ≥85% [36]. For pancreatin digestion, the viability was ≥95%, which is in good agreement with other results (>98.4%) [24]. This high viability is related to the lower concentration of pancreatin used, which should cause fewer injuries to isolated hepatocytes [24]. The viability achieved with two-step collagenase perfusion was below 85% (max 73%), which is likely related to the long-time involved in the cannulation procedure and also needs accurate technical skills. Based on the viability and cell yield results, we proceeded with the pancreatin digestion method in *S. aurata* in subsequent experiments.

#### Cell Culture Purity

The presence of blood cells and tissue debris was observed in the first extractions with the pancreatin digestion method. To improve the culture purity, we applied some modifications to the original pancreatin digestion method described by Yanhong et al. [23]. As mentioned in Section 2.2.2, these modifications included the usage of two meshes (200 and 60 µm) during the filtration process and two centrifugation steps with Histopaque^®^ (Figure 2). The usage of these two meshes improved the removal of tissue debris, and the two subsequent centrifugations (120× *g* and 140× *g*, 5 min at 4 °C) with Histopaque^®^ improved the removal of blood cells from the cell culture (Figure 2). The presence of blood cells in the resulting culture is a critical point. In the two-step perfusion method, the perfusion step aims to clean up the liver from these cells. However, several authors have suggested the low-speed centrifugation with Percoll gradient as an alternative to guarantee a pure culture of hepatocytes [24,40,41]. Taking this into account, we combined enzymatic-based dissociation with the Percoll gradient purification, using Histopaque^®^ to obtain a high-purity culture. Histopaque^®^ is a solution of polysucrose and sodium diatrizoate, adjusted to a density of 1.077 g/mL, which is commonly used in isolation and purification of blood cells [35,42]. This proved to be an adequate substitute for the perfusion method, greatly simplifying procedures.

In most cases, after this purification process, the hepatocyte culture purity was 100%, as determined by microscopic observation. The three final washing steps further improved the removal of dead cells.

### 3.2. Cell Culture Condition: Morphology, Viability, and Function

**Figure 3:**
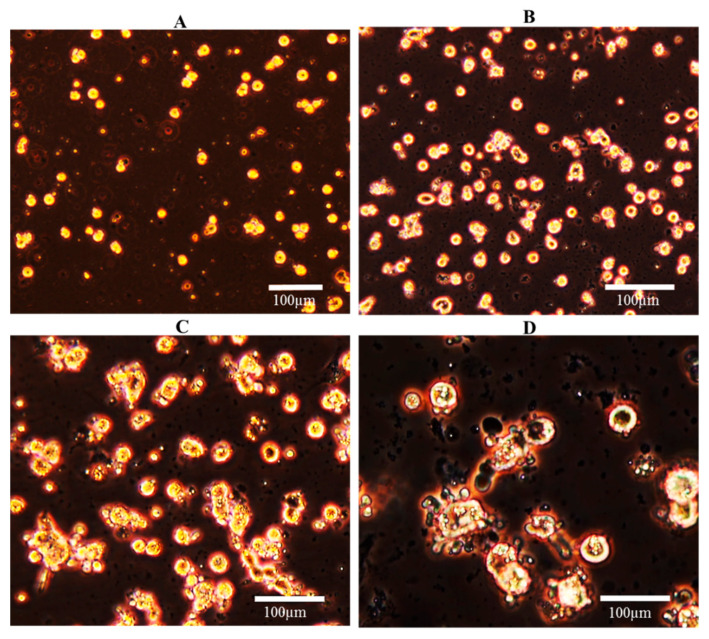
Culture of *S. aurata* primary hepatocytes isolated by pancreatin digestion with modifications (**A**) and after incubation in poly-L lysine-coated plates up to 24 (**B**), 48 (**C**), and 72 h (**D**). The observations were made under inverted light microscopy (Olympus) with a 10× objective (**A**,**B**) and a 20× objective (**C**,**D**).

The marine fish primary hepatocyte cultures obtained by the optimized pancreatin digestion method are presented in Figure 3. The culture shows ≥85% viability and 100% hepatocyte purity. The monitoring data at T_0_ to T_144_ showed that the cells were attached to a plate coated with the poly-L lysine and collagenase I coated-plate.

Immediately upon isolation, hepatocytes were seen as single separated cells, which were showing a rounded shape (Figure 3A). Twenty-four hours after isolation, and after 24 h to allow cell attachment, this initial rounded shape was changed into a triangular/polygons shape, some hepatocytes began to flatten, and the cell–cell contact was evident (Figure 3B). After 48 h in culture, cells were clustering into small groups (Figure 3C), and larger clusters were observed after 72 h, with the subsequent loss of the initial rounded appearance as the boundaries between the cells increased (Figure 3D).

Primary hepatocytes’ viability was assessed by the trypan blue exclusion and MTT assays. The trypan blue exclusion was performed at T_24_, and the results showed cell viability of 94.8 ± 14.1% for the collagen I plate and 98.1 ± 11.5 for the poly-L lysine plate (Table 2). The MTT viability test was performed at T_24_, T_48_, and T_72_. Comparing the results for plates coated with collagen I, the viability decreased slightly, reaching a minimum of the viability of 78.7 ± 0.47% at T_72_ (Table 2). Interestingly, no significant differences in cell viability were obtained when comparing the manual poly-L-lysine coating and commercial collagen I coating plates (Table 2).

**Figure 4:**
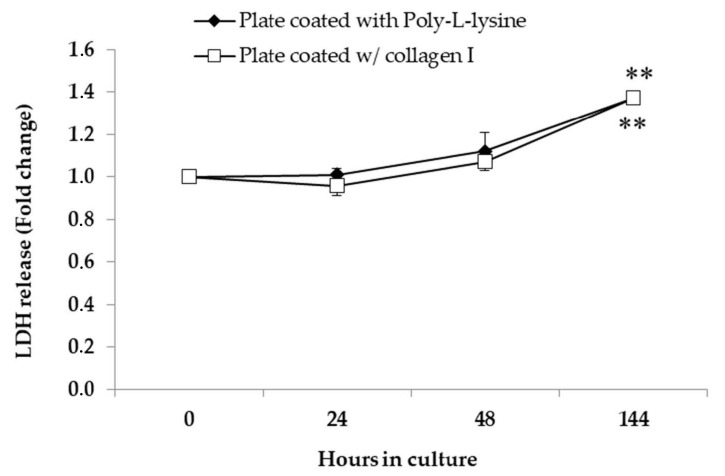
Lactate dehydrogenase (LDH) released by *S. aurata* primary hepatocytes cultured in a 24-well plate coated with poly-L-lysine and collagen I. After the overnight incubation of 10^5^ cells/well, the L-15 medium was collected at 24, 48, and 144 h to assess LDH activity. A significant difference was observed between the control and T_144_; ** *p* < 0.01.

The assessment of LDH release was performed in cell culture media and collected after T_24_, T_48_, and T_144_. Analysis of the culture supernatants showed that LDH secretion increased gradually over time, with a peak at T_144_ (Figure 4).

**Figure 5:**
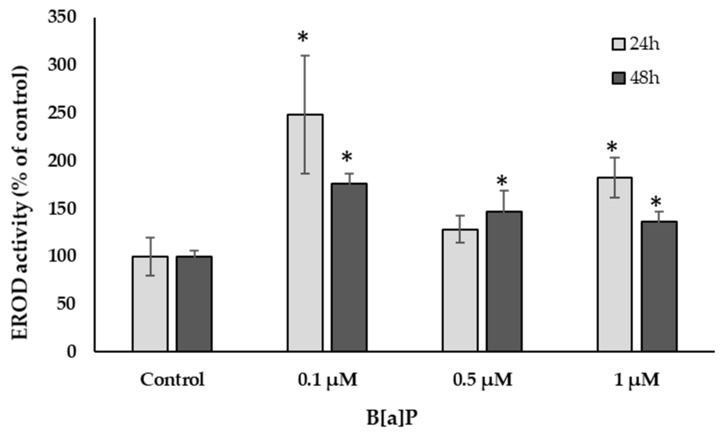
EROD activity of *S. aurata* primary hepatocytes exposed to different concentrations of Benzo(a)pyrene (B[a]P) for 24 and 48 h. Ethoxyresorufin O-Deethylase (EROD) activity is expressed relative to control cells (DMSO). Values are mean ± SE of 4 to 5 independent extractions. A significant difference was observed between the control and T_24_ and T_48_; * *p* < 0.05.

To assess cellular function, namely xenobiotic phase I metabolism, primary hepatocytes of *S. aurata* cultured on the poly-L-lysine plates were exposed to 0.1, 0.5, and 1 μM of B[a]P to assess CYPA1 activity via EROD-based assay (Figure 5). The results showed a significant increase of EROD activity at T_24_ and T_48_, relative to control cells (*p* < 0.05).

## 4. Discussion

The use of primary cells carries several challenges, including (1) the process of cell isolation, (2) the maintenance of viable and functional cells in culture, and (3) reproducibility [43,44]. Among these, the most critical challenge is to have an optimized isolation method adjusted to the demands of the investigation. Among the available methodologies, two methods were used to obtain primary hepatocytes culture from marine fish: the two-step collagenase perfusion and a common enzymatic-based dissociation by a pancreatic enzyme. Two-step collagenase perfusion combines the perfusion technique with the dissociation action of collagenase and has been successfully used to isolate primary hepatocytes for toxicological evaluation of aquatic xenobiotics purposes [14,19,45,46,47,48,49,50,51,52,53].

Although enzymatic dissociation methods are not so commonly used, the pancreatin digestion method described in the present research (Figure 1 and Figure 2) proved to be effective to isolate marine fish primary hepatocytes in terms of (1) its applicability for small/medium/large fish, (2) low cost and simple procedure, and (3) high cell yield and viability achieved (Table 1). In comparison with the two-step collagenase perfusion method, the pancreatin method proved to be very versatile, being suitable for extracting cells from smaller livers where it is difficult or impossible to apply the liver cannulation procedure, required for the two-step collagenase perfusion method. Enzymatic digestion is in general a better alternative when the model in the study is small, such as the case of zebrafish [54].

Besides these advantages, pancreatin digestion also proved to be more effective in obtaining a significantly higher number of viable cells in comparison with those obtained by the two-step collagenase perfusion method (Table 1). Using healthy *S. aurata* livers of a similar weight (*p* > 0.05), the number of viable cells obtained by pancreatin digestion was up to four-fold higher than by collagenase perfusion. Additionally, the percentage of viable cells extracted using pancreatin digestion was 30% higher than with the two-step collagenase perfusion method. This is extremely important since obtaining a high number of viable cells is the first critical point of using primary cells.

After isolation, primary cells must remain viable and functional long enough to allow successive experiments. Thus, the viability of the isolated primary hepatocytes was monitored over two to six days in culture by several tests (trypan blue exclusion test, MTT assay, and LDH release). All these assays showed a similar result (Table 2); however, MTT and LDH data had a smaller standard error, suggesting that these tests may be a more reproducible method than trypan blue exclusion by eliminating error associated with cell counting. In general, until 72 h in culture (three days after the isolation), the viability of primary hepatocytes plated in coated plates was on average >80% (Table 2). This suggests that when seeded in coated plates, either with the commercial collagen or manually with poly-L lysine, after the initial attachment, the majority of cells remain viable and stable. Furthermore, the results of the LDH release test, used to assess cell death, showed that the cells remain viable and functional for six days. Indeed, a significant increase in cell death (p < 0.01) was only observable after six days in culture (Figure 4). This result agrees with other studies, which showed a viability >85% after the first five days in culture and a significant decline for longer periods [14].

Freshwater species (such as *Oncorhynchus mykiss*, *Cyprinus carpio, Oreochromis niloticus Pelteobagrus fulvidraco*, and *Ctenopharyngodon idellus*) are the most used fish models to assess the mode of action of environmental pollutants [14,45,46,48,51,55,56,57,58,59]. However, in the context of marine environmental toxicology, marine fish primary hepatocytes are an interesting approach when assessing the effects of xenobiotics, such as polycyclic aromatic hydrocarbons (PAHs), since it increases the ecological and economic relevance to the studies. Among PAHs, B[a]P is a five-ring PAH that can be found in aquatic environments [8] and is a potent CYP1A1 inducer [4,19,30,60]. The CYP1A mediated activation of B[a]P produces DNA reactive intermediates, such as B[a]P -7,8-diol-9,10-epoxide, which can form stable DNA adducts [4,60].

Several studies have already demonstrated the toxicological response of *S. aurata* towards B[a]P exposure [30,31,32,33], including the induction of EROD activity in fish both for in vivo and in vitro models [4,19,30,60,61]. To verify the metabolic activity of primary hepatocytes obtained from *S. aurata* liver by the pancreatin digestion method, the EROD-based assay was used after cell exposure to B[a]P. In fact, after 24 and 48 h of exposure to three different concentrations of B[a]P (0.1, 0.5, and 1 µM), EROD activity increased significantly relative to control cells, showing that *S. aurata* primary cells were active and able to metabolize this xenobiotic by liver cytochrome P450 dependent-monooxygenases.

A nondependent dose–response and higher EROD activity being obtained at the lowest B[a]P concentration tested was also observed. This pattern has already been verified by Wessel et al. [61], who reported similar results for sole hepatocytes exposed to 0.1, 0.5, 1, 5, and 25 µM B[a]P concentrations. In contrast, Ferreira et al. [19], who used primary hepatocytes isolated from *Dicentrarchus labrax*, reported a dose–response result for EROD activity with a peak at a 1 µM B[a]P concentration (maximum studied concentration). However, the CYP1A1 response of fish to contaminants is known to be species specific [61]. Comparing the two exposure times, EROD activity was higher after 24 h of exposure than 48 h. This observation is in line with previous studies that reported a peak of EROD activity after 24 h of exposure of *S. aurata* treated with 20 mg/Kg of intraperitoneal B[a]P injections [30].

## 5. Conclusions

This study presented an optimized methodology for the isolation of marine fish primary hepatocytes, which can be maintained viable and functional for up to five days, an essential feature for using primary cultures in ecotoxicity testing. This optimized protocol for pancreatin digestion, besides being a good alternative to the commonly used perfusion method, proved to be a suitable method to be used in future ecotoxicological studies involving marine pollutants, such as PAHs. This is particularly relevant in the present-day context, where compound mixtures in the aquatic environment are abundant, therefore demanding fast and thorough analysis of toxicity mechanisms, using alternatives to in vivo models as stated in the 3Rs principle.

## Figures and Tables

**Table 1:** Total number of primary hepatocyte cells (range and average) isolated from the liver of *S. aurata* and *P. maxima* fish obtained by different cell isolation methods, two-step collagenase perfusion and pancreatin digestion, and their respective cell viability (%).

Method	Fish Species	Weight (g)		^1^ Number of Cells (×10^7^)	% of Viability
		Fish	Liver		
Two-step perfusion	*Sparus aurata*	234 ± 43.3	4.80 ± 1.66	1.04–2.47 $\bar{x}$ = 1.91 ± 0.77 ^a^	70.7–72.8
Pancreatin digestion	*Sparus aurata*	236 ± 41.2	4.47± 1.37	3.3–7.31 $\bar{x}$ = 4.5 ± 1.9 ^b^	95.1–100
	*Psetta maxima*	62.5 ± 8.57	0.47 ± 0.19	1.08–3.44 $\bar{x}$ = 1.97 ± 0.76 ^c^	100

^1^ Range of cell yields and the respective average of 3 ^a^, 4 ^b^, and 8 ^c^ independent assays.

**Table 2:** The viability of primary hepatocytes isolated by pancreatin digestion after T_24_, T_48_, and T_72_ in culture with different plates, no-treatment, and coated with the poly-L-lysine solution (Sigma) and collagen I (Sigma). Cell viability was evaluated by trypan blue assay (TBA) and MTT. All the data are expressed as % of T_0_ and are expressed as the mean ± SD of more than 3 independent isolations. Significant differences from the T_0_ was observed for the plates coated; * *p* < 0.05.

Viability Tests	Plate Coating	
	Plate Coated w/Poly-L Lysine	Plate Coated w/Collagen I
TBA (24 h)	98.1 ± 11.5	94.6 ± 14.1
LDH (24 h)	97.5 ± 3.4	95.5 ± 4.9
	**MTT**	
24 h	84.1 ± 8.13 *	91.8 ± 2.73
48 h	82.7 ± 4.157 *	89.0 ± 2.93 *
72 h	82.7 ± 8.15 *	78.7 ± 0.47 *

## Data Availability

The data presented in this study are available on request from the corresponding author.
